# Intracellular Protein Shuttling: A Mechanism Relevant for Myelin Repair in Multiple Sclerosis?

**DOI:** 10.3390/ijms160715057

**Published:** 2015-07-03

**Authors:** Peter Göttle, Patrick Küry

**Affiliations:** Department of Neurology, Medical Faculty, University of Düsseldorf, 40225 Düsseldorf, Germany; E-Mail: Peter.Goettle@uni-duesseldorf.de

**Keywords:** multiple sclerosis, myelin repair, nucleocytoplasmic shuttling, oligodendrocyte, differentiation inhibitors, transcription factors, remyelination

## Abstract

A prominent feature of demyelinating diseases such as multiple sclerosis (MS) is the degeneration and loss of previously established functional myelin sheaths, which results in impaired signal propagation and axonal damage. However, at least in early disease stages, partial replacement of lost oligodendrocytes and thus remyelination occur as a result of resident oligodendroglial precursor cell (OPC) activation. These cells represent a widespread cell population within the adult central nervous system (CNS) that can differentiate into functional myelinating glial cells to restore axonal functions. Nevertheless, the spontaneous remyelination capacity in the adult CNS is inefficient because OPCs often fail to generate new oligodendrocytes due to the lack of stimulatory cues and the presence of inhibitory factors. Recent studies have provided evidence that regulated intracellular protein shuttling is functionally involved in oligodendroglial differentiation and remyelination activities. In this review we shed light on the role of the subcellular localization of differentiation-associated factors within oligodendroglial cells and show that regulation of intracellular localization of regulatory factors represents a crucial process to modulate oligodendroglial maturation and myelin repair in the CNS.

## 1. Introduction

The central nervous system (CNS) is composed of two major classes of cells—neurons and glial cells—the latter of which can be subdivided into astrocytes, microglia and oligodendrocytes. While neurons provide the basis for signal transduction and information processing, glial cells account for a wide range of specific functions. Oligodendrocytes generate myelin sheaths surrounding axons, and they are therefore referred to as myelinating glial cells. Myelin sheaths are imperative for stabilization, protection, and electrical insulation of axons, which enables accelerated electrical signal propagation [1,2,3]. This intimate interaction between axons and oligodendrocytes is highly vulnerable and can become functionally impaired or damaged upon traumatic CNS injury or in inflammatory demyelinating diseases, such as multiple sclerosis (MS). This disease features damage to myelin sheaths and oligodendrocytes owing to autoimmune- as well as neurodegenerative processes, leading to sustained myelin loss, impaired electrical signal transduction, and eventually axonal degeneration. MS is by far one of the most common demyelinating neuroinflammatory diseases, affecting approximately 2.5 million people worldwide [4]. Its etiology is unknown, and both genetic and environmental factors are assumed to contribute to the pathogenesis [5]. Due to the restricted capacity of the CNS to regenerate and repair damaged tissue, traumatic injuries or neurological diseases generally result in permanent damage, functional loss, and persisting disabilities. Interestingly, this diminished repair capacity not only relates to neurons and their axons, but also to mature and fully differentiated oligodendrocytes, which cannot compensate for myelin loss because they usually degenerate in diseased brain areas [6,7].

However, demyelination can be counteracted to a certain extent via recruitment and activation of resident adult oligodendroglial precursor cells (OPCs) [8]. These cells comprise 5%–8% of the total cell population in the adult human and rodent CNS throughout the gray and white matter [9,10,11,12], and they can be identified by the expression of markers such as neural/glial antigen 2 (NG2) and platelet-derived growth factor receptor α [13,14]. Similar to perinatal OPCs, this fraction of adult OPCs also expresses transcription factors such as Nkx2.2, Ascl1/Mash1, Olig2, and MyT1 under physiological and pathological conditions [15,16,17,18,19,20,21]. Remyelination-related processes such as activation, recruitment, and differentiation [22,23] are tightly regulated by a number of extrinsic and intrinsic factors that act either as inhibitors or activators [24,25]. Upon demyelination, OPCs switch from an essential inert state to a mitotically active phenotype, which is accompanied by the up-regulation of oligodendrogenic genes, such as *Olig1/2* and *Nkx2.2* [13,15,26]. Following OPC recruitment, cellular differentiation comprises contact with the demyelinated axons and expression of myelin genes and proteins, as well as a process of wrapping around axons with subsequent compaction to generate myelin sheaths [22,27].

Remyelination can be very effective in experimental *in vivo* models, such as those based on cuprizone-mediated demyelination or upon direct injection of toxins such as lysolecithin or ethidium bromide [28]. Nevertheless, myelin repair in MS varies between patients, lesions, and disease stages, and the efficiency of this endogenous repair process remains generally low, thus contributing to permanent deficits and dysfunctions. To a certain extent this decline correlates with failure of OPCs to successfully generate new myelinating cells. Although the underlying reasons are not yet fully understood, several lines of evidence point to the presence of multiple differentiation inhibitors that specifically constrain the glial regeneration potential [25,29,30,31]. To treat existing MS lesions and to support myelin repair therapeutically, it is therefore important to exploit features of the naturally occurring repair process and to identify rate-limiting factors and signals. The aim of this review article is to show the extent to which regulated subcellular protein distribution is involved in oligodendrogenesis, and how this could be used to devise new interventional strategies.

## 2. Intracellular Protein Shuttling—A Mechanism Involved in Neurodegenerative Diseases?

Intracellular protein shuttling is essential for protein function and for functional and spatial diversity [32] because transport to specific subcellular sites can determine access to specific substrates or interaction partners, and also allows incorporation into functional biological mechanisms/pathways. This transport is highly regulated and occurs through nuclear pore complexes (NPCs) that allow ions, small molecules, and proteins smaller than 40 kDa to cross the nuclear envelope [33]. Larger proteins are actively shuttled by two opposing nucleocytoplasmic transport receptors termed karyopherins in a signal-mediated manner. Importins compose a family of 16 members, including α and β transportin, that are responsible for transport from the cytoplasm to the nucleus, whereas exportins comprise six family members, including Crm1/XPO/exportin1, which is involved in nuclear export [34,35,36]. These transport receptors mediate both translocation processes by recognizing specific nuclear localization and export signals (NLSs and NESs, respectively) and thus directing the distribution of cargo proteins [37,38].

**Figure 1:**
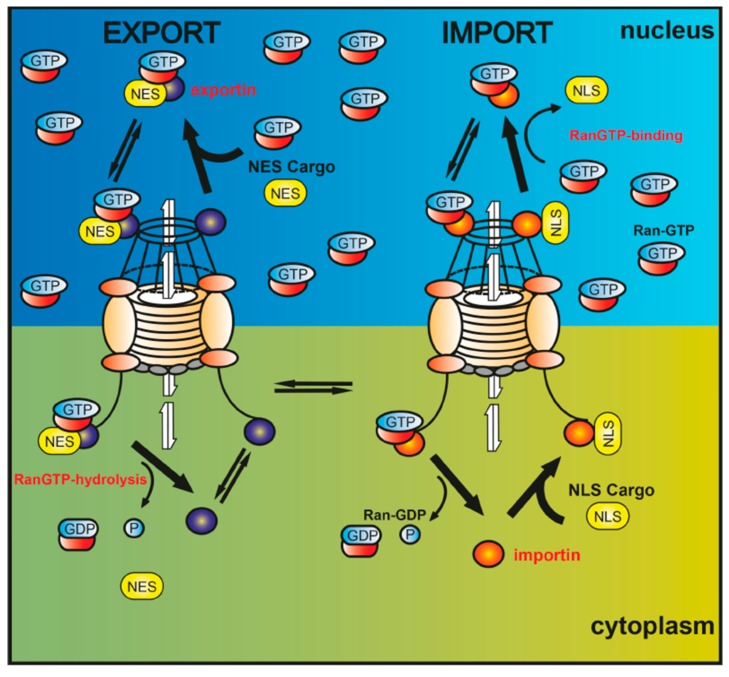
Nuclear import of cargo proteins implicates the recognition of a nuclear localization sequence (NLS) by importin receptors. In the nucleus, importins are dissociated from the cargo through the binding of Ran-GTP. Nuclear guanine-nucleotide exchange factors (GEFs) maintain elevated nuclear Ran-GTP concentration levels. On the other hand, translocation to the cytoplasm is facilitated by a nuclear export sequence (NES), recognized by exportin receptors such as CRM1 and further interacting with Ran-GTP. Upon translocation of the export complex to the cytoplasm, the cargo dissociates from the complex by means of GTPase-activating proteins (GAP)-mediated hydrolyzation of Ran-GTP to Ran-GDP.

Translocation through the NPC occurs after receptor-cargo complexes interact with NPC proteins, followed by interaction with Ras-related nuclear protein guanosine triphosphate (Ran-GTP) and subsequent cargo release to the dedicated subcellular compartment [39]. The active protein import across the NPC is powered by a nucleocytoplasmic Ran-GTP gradient and requires the hydrolysis of two GTPs. Nuclear localized guanine-nucleotide exchange factors (GEFs) maintain an elevated nuclear Ran-GTP concentration because they catalyze the exchange of guanosine diphosphate GDP to GTP on Ran molecules. Upon binding of an importin receptor to a cargo protein with an NLS sequence, this receptor-cargo complex is directed towards the NPC and passes through. The cargo is released in the nucleus by the binding of Ran-GTP to the importin receptors and subsequent displacement of the cargo. The importin/Ran-GTP complex then diffuses back to the cytoplasm where GTPase-activating proteins (GAPs) hydrolyze GTP to GDP, which leads to the release of importins and thus the availability for new import processes. However, nuclear cargo proteins with an NES sequence can bind to a previously formed exportin (*i.e.*, chromosome region maintenance 1, CRM1; also referred to as exportin1 or Xpo1) and Ran-GTP complex, and this heterotrimeric complex can then diffuse through the NPC into the cytoplasm. On the cytoplasmic side, the cargo dissociates from the complex by means of GAP-mediated hydrolyzation of GTP to GDP. CRM1 and Ran-GDP then diffuse back to the nucleus where GEFs exchange GDP to GTP (Figure 1).

Because nuclear localization of proteins such as transcription factors is key to their function, and an adequate localization of signaling proteins close to their downstream targets can be imperative for many signal transduction pathways, unbalanced nucleocytoplasmic shuttling can result in their inactivation. Undesired gain-of-functions can thus cause a variety of cellular malfunctions related to signaling, metabolic or structural properties [40]. Nucleocytoplasmic transport failure has in fact been implicated in several neurodegenerative diseases and conditions, such as amyotrophic lateral sclerosis (ALS) [41,42,43], Alzheimer’s disease [44], Huntington’s disease [45], traumatic injury [46], and MS [47,48]. For most of these disorders, neurodegeneration and axonal damage correlate with mislocation of proteins, such as histone-1 and β-catenin in the anterior horn cells of mutant Cu/Zn-superoxide dismutase (G93A) transgenic mice, an animal model for ALS, or in hippocampal neurons in Alzheimer’s disease, revealing cytoplasmic accumulation of the nuclear transport factor 2 [42,44]. In demyelinating diseases such as MS, particularly in the context of previously discussed endogenous repair activities, cells of the glial lineage are of primary interest. They have also been shown to be affected by impaired/dysregulated nucleocytoplasmic translocation, which will be presented in the next section in detail.

## 3. Nucleocytoplasmic Translocation Activities of Myelinating Glial Cells

Even though the restricted capacity of the CNS to regenerate myelin sheaths is linked to different regulatory factors/pathways, a broad range of regulators share common features. Not only do their expression and presence appear to limit glial maturation, but their subcellular localization within the oligodendroglial cells is assumed to constrain the regeneration process as well. Hence, precise and timely nucleocytoplasmic transport of these regulators across the nuclear membrane represents an essential mechanism for controlling their activity. For this reason, nuclear transport could be considered as a putative therapeutic target with potential implications for neurodegenerative diseases. This review therefore focuses on aberrant/dysregulated nucleocytoplasmic protein shuttling within oligodendroglial cells, its implication/contribution to the restricted capacity of the CNS to repair damaged tissue, and the description of possible molecular targets that could be used to develop therapeutic strategies.

### 3.1. Cell Cycle-Associated Proteins

In order to generate functional oligodendrocytes, cell cycle progression and differentiation must be tightly controlled. The shift between proliferation and differentiation depends on regulatory complexes involving cyclins, cyclin-dependent kinases (CDK), and cyclin-dependent kinase inhibitors (CDKIs) as well as phosphorylation-dephosphorylation events, all responding to intracellular and extracellular signals and influencing their expression levels and intracellular localization [49]. Proliferating oligodendroglial cells feature elevated cyclin E and D; increased CDK2, -4, and -6 protein levels; and higher kinase activities of both CDK4/6-cyclin D and CDK2-cyclin E complexes as compared to cells permanently withdrawn from the cell cycle [50]. In addition, Frederick and colleagues demonstrated that nuclear accumulation of cyclin D1 within OPCs is promoted upon fibroblast growth factor -2 and insulin-like growth factor-I stimulation [51] and enhanced S-phase entry [52].

**Figure 2:**
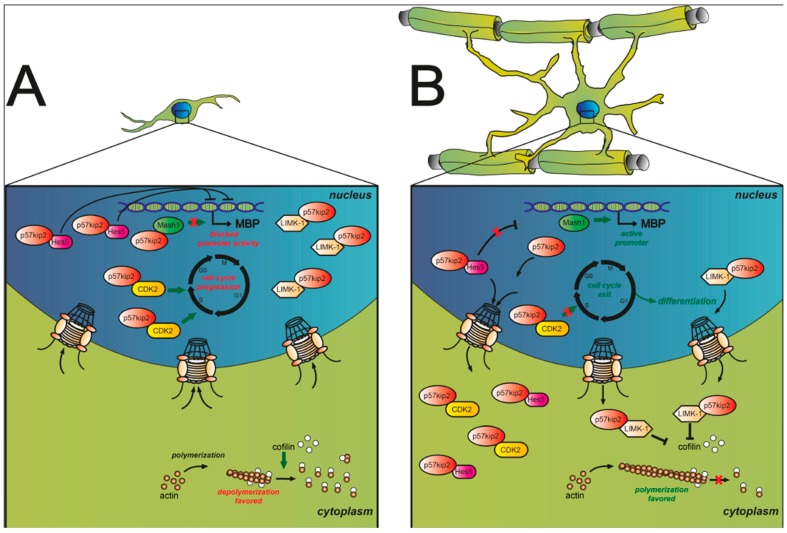
(**A**) In early stages of oligodendroglial precursor cell (OPC) differentiation p57kip2 is predominantly located in nuclei. Kinase binding partners such as LIM Domain Kinase 1 (LIMK-1) and CDK2 appear to colocalize with p57kip2. Nuclear presence of CDK2 results in maintenance of cell cycle progression, while nuclear LIMK-1 is unable to inhibit cytoplasmic cofilin, a negative regulator of actin filament turnover. As a result actin depolymerization is enhanced. Moreover, interactions of p57kip2 with the transcription factors Ascl1/Mash1 and Hes5 appear to control target gene transcription. Upon p57kip2 binding Ascl1/Mash1’s transcriptional activity was found to be blocked while Hes5 nuclear accumulation appears to be enforced by p57kip2 where it is supposed to interfere with myelin gene expression; (**B**) Mature oligodendroglial cells exhibit high cytoplasmic levels of p57kip2. Nuclear export of p57kip2 leads to dissociation from its binding partner Ascl1/Mash1 and results in myelin gene activation by this transcription factor. Furthermore, CDK2 and Hes5 translocate from the nucleus to the cytoplasm along with p57kip2 and are thus rendered functionally inactive. In addition, enhanced cytoplasmic levels of LIMK-1 can affect actin filament turnover. Cytoplasmic LIMK-1 is phosphorylating cofilin, which in turn interferes with its capacity to depolymerize actin filaments. Subsequently, this process of cytoplasmic trapping of inhibitory components by the p57kip2 protein, allows terminal differentiation to proceed.

Furthermore, nuclear activity of CDKs, especially of CDK2 maintaining the G1/S-phase checkpoint together with cyclin E, was shown to be necessary to retain cell cycle progression in mammals [53]. Loss of CDK2, on the other hand, enhanced cell cycle exit and glial maturation, whereas active nuclear exclusion was found during the differentiation process in differentiating OPCs [54,55] (Figure 2, Table 1). Upon lysolecithin (LPC)-induced focal demyelination in adult mice, loss of CDK2 was shown to attenuate cell proliferation and to accelerate differentiation and remyelination [54].

CDK5 absence led to nuclear translocation of the transcription factor FOXO1 and subsequently facilitated neuronal cell death [56]; it also reduced myelin mRNA and myelination and delayed differentiation in oligodendroglial cells [57]. Because retrograde intracellular transport depends on CDK5 activity [58], loss of function and thus decreased OPC maturation have been proposed to largely reflect perturbed transport of mRNA, including that coding for myelin basic protein (MBP). Of note, intracellular transport of RNAs will be further described here in Section 3.5. Nuclear expression of CDK5 is increased following oligodendroglial differentiation [57,59], and phosphorylation of the focal-adhesion-associated protein paxillin as well as of WASP family verprolin-homologous proteins WAVE1 and WAVE2 by CDK5 has been proposed to regulate OPC migration, differentiation, and myelination [59,60,61]. This suggests that in oligodendroglial cells, CDK5’s primary function appears to be the modulation of cellular differentiation rather than cell division (Table 1).

E2F1, a member of the E2F family of transcription factors, plays a critical role in coordinating early cell cycle progression; in its unbound state, it directs the transcription of genes required for entry into the S phase [62]. Unbound nuclear E2F1 is dependent on the phosphorylation state of retinoblastoma protein Rb, which sequesters E2F1 in its unphosphorylated state [63]. However, increased Rb phosphorylation resulting from up-regulated CDK/Cyclin complexes leads to E2F1 release [64], and recent studies revealed that OPC differentiation *in vitro* and *in vivo* is accompanied by nuclear export of E2F1 [65]. E2F1 was identified as a key transcription factor modulating the expression of chromatin components in OPCs during the transition from proliferation to differentiation. Its nucleocytoplasmic shuttling was suggested to be required to dampen the E2F1-driven pattern of expression, thus allowing OPC differentiation to occur (Table 1).

CDKIs negatively regulate cell cycle progression by binding and inactivating cyclin/CDK complexes. Based on their CDK binding specificity, CDKI proteins fall into two subfamilies-namely, the inhibitor of kinase 4 (INK4) family and the CDK interacting protein/kinase inhibitory protein (Cip/Kip) family [66]. The INK4 family includes p16INK4a, p15INK4b, p18INK4c, and p19INK4d, all of which competitively bind to CDK4 and CDK6 and prevent complex formation with cyclin D and block G1/S-phase transition. Cip/Kip family members comprise p21cip1, p27kip1, and p57kip2 interacting with CDK2, -4 and -6 [66,67] (Table 1). p27kip1 and p21cip1 were demonstrated to be important regulators of OPC proliferation and differentiation initiation because their nuclear expression increased during differentiation or during reversible cell cycle arrest in G1 caused by neuronal signals [68,69]. Altered subcellular localization of p27Kip1 was shown to act as an intrinsic timer for oligodendroglial development that was found to progressively increase during rodent optic nerve development in a time-dependent manner as well as in purified OPC cultures [70,71]. Overexpression and subsequent nuclear accumulation of p27kip1 were then shown to terminate proliferation, enhance MBP expression, and promote terminal differentiation [72,73,74]. Likewise upon CNS injury, mitogen signals induced p27kip1 phosphorylation on serine-10, leading to CRM1-mediated nuclear export that facilitated cell cycle progression, especially in microglia and astrocytes [75]. Upon LPC-induced demyelination, loss of p27kip1 also increased the proliferative response of OPCs to injury [76].

In proliferating OPCs, the expression of the tumor suppressor p53 is low, and the protein is likely not functional based on its cytoplasmic localization [77]. Translocation to the nucleus occurs upon mitogen removal, and p53 is then involved in the onset of OPC differentiation by stimulating p21cip1 expression, followed by the inhibition of CDK2-cyclin E complexes [77]. Similarly, overexpression and nuclear accumulation of p18INK4c have also been shown to prevent OPC proliferation and to accelerate differentiation onset [78,79]. However, *in situ* analysis of active MS lesions revealed increased expression of p53 in apoptotic oligodendrocytes suggesting it to be a critical pro-apoptotic effector [80]. Accumulation of p53 resulted in up-regulation of death receptors, the ligands of which are present in the inflammatory milieu of active MS lesions (Table 1).

As opposed to the general view of Cip/Kip proteins functioning exclusively as inhibitors of cyclin/CDK activities, increasing evidence suggests that they exert additional cellular functions outside the nucleus, such as regulation of cellular processes [81,82]. In this regard, we demonstrated that the cyclin-dependent inhibitor p57kip2 negatively affects myelinating glial cell differentiation in the peripheral and central nervous system and that it controls glial fate decision by adult neural stem cells [55,83,84,85]. Despite Cip/Kip family members being highly homologous in their N-terminal CDK-binding and C-terminal domains, p57kip2 features a large central proline-rich region distinct from all other CDKIs that possibly has unique functions not shared by p21cip1 and p27kip2 [86,87]. This proline-rich region allows p57kip2 to bind LIMK-1. The nuclear presence of p57kip2 therefore prevents LIMK-1 from phosphorylating cytoplasmic cofilin and thus favors actin filament destabilization and disassembly [88,89]. The p57kip2 protein, however, translocates spontaneously in differentiation-competent OPCs, and this subcellular translocalization process is key to its inhibitory role [55]—that is, promoting myelin expression, higher morphological phenotypes, and enhanced myelination *in vitro* (Figure 2). In a recent study of established MS lesions, no evidence of a p57kip2 translocation in white matter OPCs could be found, suggesting a functional implication, in that prevented or slowed myelin repair could suffer from averted p57kip2 export [90].

**Table 1:** Cell cycle-associated key molecules.

Molecules	Major Function in OPCs	Role in MS/MS-Models	References
CDK5	migration, differentiation and myelination, mRNA transport	–	Miyamoto *et al.*, 2007/2008 [59,60]; Yang *et al.*, 2013 [57]; Zhou *et al.*, 2015 [56]
CDK2	cell cycle progression	LPC: alters adult OPCs renewal, differentiation, remyelination	Malumbres *et al.*, 2005 [53]; Caillava *et al.*, 2011 [49]; Göttle *et al.*, 2015 [55]
E2F1	modulates chromatin components during transition from proliferation to differentiation	–	Magri *et al.*, 2014 [65]
p21cip1	proliferation, differentiation	–	Ghiani *et al.*, 1999 [50]
p53	proliferation, differentiation	MS lesions: apoptosis	Eizenberg *et al.*, 1996 [77]; Wosik *et al.*, 2003 [80]
p27kip1	proliferation	LPC: proliferative response	Crockett *et al.*, 2005 [76]; Raff *et al.*, 2007 [70]; Durand *et al.*, 1997/1998 [71,74]; Miskimis *et al.*, 2002 [72]; Tamaki *et al.*, 2004 [73]
p57kip2	glial fate decision, differentiation	MS lesions: myelin repair	Kremer *et al.*, 2009 [85]; Jadasz *et al.*, 2012 [84]; Pfeifenbring *et al.*, 2013 [90]; Göttle *et al.*, 2015 [55]

Abbreviations: lysolecithin (LPC), oligodendroglial precursor cells (OPCs), Multiple Sclerosis (MS).

### 3.2. Transcriptional Regulators

Oligodendroglial lineage transcription factors Olig1 and Olig2 are basic helix-loop-helix (bHLH) proteins and important positive regulators of OPC differentiation [91] (Table 2). Their expression is induced by the secreted protein sonic hedgehog (Shh) during CNS development [92]. Signaling through the canonical Gli pathway by binding of Shh to the Ptchd1 receptor stops the inhibition of the transmembrane protein Smoothened and allows the transcriptional activators Gli2/3 to translocate into the nucleus, where they induce the expression of Shh-target genes [93]. Olig1/2 expression is required for both lineage determination and differentiation to proceed [94,95], as shown by Olig1/2-deficient mice failing to develop progenitor cells [95,96]. Interestingly, although both proteins are localized in the nucleus during development, they exhibit different localization patterns in the adult. While Olig2 remains in the nucleus, Olig1 is transferred to the cytoplasm in mature oligodendrocytes [17]. Apart from facilitating myelin gene expression [97,98], Olig1 appears to have a second function; upon phosphorylation of serine 138, it translocates to the cytoplasm and promotes membrane expansion and maturation of oligodendrocytes [99,100]. In a cuprizone-mediated demyelination model and MS patient tissues, however, Olig1 relocated to the oligodendroglial nucleus, which suggested that it promotes the remyelination process [17,101,102]. Similarly, when neural stem cells (NSCs) generated GFAP-positive astrocytes in culture, Olig2 disappeared from the nucleus and appeared in the cytoplasm [103], raising the possibility that the nucleocytoplasmic translocation of Olig2 might be involved in astrogenesis [104]. This possibility is further supported by Olig2 translocation mediating brain injury-induced differentiation of NG2-positive progenitor cell to GFAP-positive astrocytes in the adult [105].

The achaete-scute complex homolog 1 (Ascl1/Mash1) protein encodes another transcriptional regulator important for oligodendroglial specification, differentiation, and myelination [106,107,108] (Table 2). Ascl1/Mash1 operates in genetic interactions with Olig2 during OPC specification, and loss of Mash1 function affects oligodendrogenesis. Ascl1/Mash1 activity is also required for proper differentiation into oligodendrocytes as it is known to regulate MBP promoter activity [108,109]. During remyelination, both upon LPC-induced demyelination of the corpus callosum as well as in MS lesions, Ascl1/Mash1 activity was up-regulated along with increased oligodendrogenesis [108]. A direct physical interaction with the p57kip2 protein occurs, and as a consequence of this binding, Ascl1/Mash1 transactivation properties are reduced. However, upon cytoplasmic translocation of p57kip2, Ascl1/Mash1 remains nuclear and can exert its full gene regulatory potential [55] (Figure 2). This binding behavior differs from LIMK-1 and CDK2 and suggests multiple signaling pathways control complex assembly and disassembly around p57kip2.

The zinc-finger myelin transcription factor 1 (Myt1) is a DNA-binding protein that regulates proteolipid (PLP) transcription. [19]. Interestingly, its nuclear accumulation was shown to be primarily required for OPC maturation because dominant negative Myt1 blocked OPC maturation and slightly inhibited proliferation [19,110]. In OPCs Myt1 accumulated in the nucleus and continued to be expressed in cells transcribing PLP, whereas in mature oligodendrocytes it was restricted to the cytoplasm [111]. Myt1 was suggested to play a role in oligodendroglial precursor cell responses to demyelination. Myt1 is localized in nuclei of OPCs in MS lesions and increased numbers of Myt1 positive OPCs were observed following murine hepatitis virus strain A59 (MHV) induced de- and remyelination [19].

Inhibitor of differentiation (Id) proteins are non-DNA-binding transcriptional regulators, located downstream of the BMP signaling, that exert strong inhibitory effects on oligodendroglial differentiation. Nuclear accumulation of Id2 and Id4 results in complex formation with Olig1, Olig2, or Ascl1/Mash1 proteins for subsequent sequestration [112,113]. These proteins translocate to the cytoplasm when spontaneous OPC differentiation proceeds, probably as a result of mitogen deprivation [114], whereas Id2 and Id4 overexpression dominantly blocks myelin gene expression [109].

In 2009 the G-protein coupled receptor 17 (GPR17) was found to be transiently expressed during oligodendrogenesis and to interfere with oligodendroglial differentiation via Id2 [113,115] (Table 2). In pathophysiological settings, such as MS or MOG_35–55_ induced experimental autoimmune encephalomyelitis (EAE), GPR17 appears to be up-regulated [113], and its overexpression in mice or cultured OPCs has been shown to inhibit the oligodendroglial maturation by enforcing nuclear localization of Id2/4 [113].

The nuclear presence of members of the Wnt/β-catenin signaling cascade in MS lesions, as well as recent observations on inhibited myelination and differentiation of oligodendrocytes following constitutive expression of β-catenin, suggests an active role regarding remyelination failure [116,117,118]. Upon binding of Wnt to the transmembrane receptor Frizzled (FZD) in complex with the co-receptor low-density lipoprotein receptor-related protein (LRP), the cytoplasmic protein Disheveled (Dsh) was activated, preventing proteosomal degradation of β-catenin via inhibition of the glycogen synthase kinase 3-β (GSK3β) [119,120]. In this context, pharmacological inhibition of GSK3β was demonstrated to promote nuclear translocation of β-catenin *in vivo* [121]. Stabilized β-catenin translocates into the nucleus and subsequently interacts with transcription factors. One such factor is T-cell factor/lymphocyte enhancer factor 4 (Tcf4/Lef), with which β-catenin forms a complex that has been found to impair development of oligodendrocytes by repressing Olig2 expression [117,122,123]. In human brains Tcf4 protein was expressed during development and in early MS lesions, where it colocalized with Olig2 in oligodendrocyte lineage cells [117,118]. Interestingly, prevention of β-catenin translocation to the nucleus was demonstrated to promote remyelination and myelination [124]. In the absence of β-catenin, TCF/LEFs assembled alternative complexes with alternate transcriptional co-repressors, such as Groucho/TLE or SMAD transcription factors [125,126].

SMADs are downstream effectors of bone morphogenic proteins (BMPs), secreted proteins that form the largest subclass of the transforming growth factor-β (TGFβ) superfamily and participate in multiple steps during nervous system development [127] (Table 2). Binding to the corresponding BMP receptors leads to phosphorylation of the receptor-regulated transcription factors SMAD1/5/8 [128]. Oligodendroglial cells express BMP4 and all three BMP receptors [129], and receptor activation has been shown to inhibit oligodendroglial differentiation and promote astroglial differentiation [129,130]. On the other hand, inhibited BMP signaling during experimental demyelination (cuprizone) was shown to promote mature oligodendrocyte regeneration and myelin repair [131]. During demyelination increased activity of BMP signaling could also be observed in the injured rat spinal cord [132,133], following local chemical demyelination [134], in EAE [135] as well as in MS lesions [136]. As a result evaluated levels of phosphorylated SMAD1/5/8 could be detected [131]. BMP-mediated phosphorylation of SMAD1 resulted in nuclear translocation and formation of a complex with the transcription factors Stat3 and p300, leading to direct activation of the GFAP promoter [137]. Furthermore, TGFβ-induced phosphorylation of SMAD2/3 promoted formation of a heterotrimeric complex with SMAD4, subsequent translocation to the nucleus, and initiation of TGFβ target genes [138]. In contrast, MAPKs catalyzed inhibitory phosphorylation in the SMAD1 linker region that resulted in nuclear exclusion of SMAD1, and thapsigargin-mediated increase of intracellular Ca^2+^ concentrations were found to reduce SMAD2 nuclear translocation [139,140].

The genes encoding bHLH transcription factors hairy and enhancer of split-1 and -5 (Hes1 and Hes5) have been implicated as critical targets of the Notch signaling pathway, that was shown to be involved in oligodendroglial differentiation and also in T helper (Th) cell activation and differentiation [141,142] (Table 2). Notch receptors are expressed by oligodendroglial cells [47,143] and can be activated by the Delta and Jagged1 ligands expressed by neurons and astrocytes, and they have been found to be up-regulated in experimental models such as EAE and Theiler’s murine encephalomyelitis virus induced demyelinating disease (TMEV-IDD) as well as in MS brains [141,144,145,146]. Blockade of the Notch signaling pathway resulted in a decrease of interferon-γ, interleukin-4 or interleukin-10 producing CD4+ T cells and increased numbers of interleukin-17 producing CD4+ T cells in the spinal cords of TMEV-IDD mice resulting in a significant suppression of the disease progression [146]. Within and around active MS plaques lacking remyelination, Jagged1 was expressed at high levels by hypertrophic astrocytes, whereas Notch1 and Hes5 localized to cells with an immature oligodendrocyte phenotype [144]. In response to Jagged1, Notch signaling is initiated by proteolytic cleavage, driven by ADAM metalloprotease and a γ-secretase complex, and nuclear translocation of the Notch intracellular domain (NICD). In the nucleus, NICD interacts with the DNA-binding protein CSL (RBP-Jk) to activate Hes1 and Hes5 [147]. Nuclear expression of Hes5 is very prominent in immature oligodendrocytes, and it has been found to prevent terminal oligodendroglial differentiation and myelination [144,148]. Hes5 mediates its inhibitory effect in two ways. First, by sequestering transcriptional activators such as Ascl1/Mash1 or Sox10, and second, by directly binding to the regulatory promoter regions of myelin genes, such as MBP, thereby blocking transcription, and epigenetically modifying chromatin [148]. Of note, the negative impact of Hes5 appears to be overcome in spontaneously differentiating OPCs by means of nuclear export, which has been found to depend on direct interaction with p57kip2 [55] (Figure 2).

**Table 2:** Key transcriptional regulators.

Molecules	Major Function in OPCs	Role in MS/MS-Models	References
Olig1/2	lineage determination, differentiation	cuprizone, MS lesion: activation of OPCs, remyelination	Arnett *et al.*, 2004 [17]; Balabanov *et al.*, 2005 [101]; Cheng *et al.*, 2015 [102]
Ascl1/Mash1	OPC specification, differentiation, myelination	LPC, MS lesions: oligodendrogenesis, promoted remyelination	Gokhan *et al.*, 2005 [109]; Parras *et al.*, 2007 [106]; Göttle *et al.*, 2015 [55]; Sugimori *et al.*, 2008 [107]; Nakatani *et al.*, 2013 [108]
GPR17 Id2/4	inhibited differentiation	LPC, MS lesions: diminished remyelination	Lecca *et al.*, 2008 [115]; Chen *et al.*, 2009 [102]
SMAD	OPC specification, inhibited differentiation	cuprizone, MS lesion: diminished remyelination, astrogenesis/gliosis	Grinspan *et al.*, 2000 [130]; Kondo *et al.*, 2004 [129]; Setoguchi *et al.*, 2004 [104]; Fuller *et al.*, 2007 [134]; Ara *et al.*, 2008 [135]; See *et al.*, 2009 [127]; Sabo *et al.*, 2011 [131]; Wang *et al.*, 2011 [132]
Notch Hes1/5	inhibited differentiation	EAE, TMEV-IDD, MS lesion: activation and differentiation of T-helper cells, diminished remyelination	Jarriault *et al.*, 1998 [141]; Wang *et al.*, 1998 [132]; John *et al.*, 2002 [144]; Hu *et al.*, 2003 [143]; Liu *et al.*, 2006 [148]; Elayman *et al.*, 2009 [145]; Nakahara *et al.*, 2009 [47]; Tsugane *et al.*, 2012 [146]

Abbreviations: lysolecithin (LPC), oligodendroglial precursor cells (OPCs), Multiple sclerosis (MS), experimental autoimmune encephalomyelitits (EAE), Theiler’s murine encephalomyelitis virus (TMEV)-induced demyelinating disease (TMEV-IDD).

### 3.3. Posttranscriptional and Posttranslational Factors

Acetylation and deacetylation of histones essentially contribute to the regulation of gene expression and are catalyzed by histone acetyltransferases or histone deacetylases (HDACs), respectively. Acetylation is associated with active open chromatin and an initiated gene expression, whereas deacetylation blocks the expression of, for example, inhibitory transcription factors such as Hes5, Id2 and Id4, Tcf7l2 and Tcf4 and can thus promote myelination [117,149,150]. However, Hes5 can form repressive complexes with HDACs, and insufficient recruitment of HDACs to promoter regions of such inhibitory transcriptional factors can consequently result in their continuous expression. Interestingly, this reflects the situation in cuprizone mediated demyelination of aged rodent brains, where HDAC recruitment is inefficient allowing the accumulation of transcriptional inhibitors and reduces their remyelination potential compared to younger animals [150]. Of note, administration of pharmacological HDAC inhibitors (HDACi) during cuprizone treatment or after MOG_35–55_ induced EAE was able to recapitulate defective remyelination *in vivo* [150,151,152]. This is in accordance with the recent finding of Ye and colleagues demonstrating that oligodendroglial differentiation requires transcriptional co-repressors HDAC1 and HDAC2. Depletion of both genes resulted in stabilization and nuclear translocation of β-catenin, which, as already discussed, prevents *Olig2* expression and thus oligodendroglial differentiation. It was therefore assumed that HDAC1 and HDAC2 compete with β-catenin for Tcf4/Lef interaction to regulate downstream genes involved in oligodendroglial differentiation [123]. Studies on MS tissue samples suggested that histone deacetylation is a process rather restricted to early disease stages, the efficiency of which decreases with disease duration [153].

As yet another structural protein modification process, citrullination of arginine residues is catalyzed by peptidylarginine deiminases (PADs). In normal-appearing white matter of MS patients and in a ND4 transgenic mouse model for chronic progressive primary demyelination, TNFα-dependent citrullination of histones by PAD4 was observed [154]. Enhanced citrullination of MBP was shown to destabilize myelin sheaths in MS. High citrullination of histones, as a consequence of nuclear PAD4, is assumed to lead to irreversible changes in oligodendroglial chromatin organization and may contribute to oligodendroglial cell apoptosis in MS [154].

The ten-eleven translocation (TET) family of methylcytosine dioxygenases catalyzes oxidation of 5-methylcytosine (5mC) to 5-hydroxymethylcytosine (5hmC) and promotes DNA demethylation. TET proteins have been demonstrated to regulate promoter activities in embryonic stem cells [155]. Three TET family members (TET1, TET2, and TET3) were found to be critical gene expression modifiers by means of DNA-methylation during oligodendroglial differentiation, and all were characterized by unique subcellular and temporal expression patterns [156]. TET1 protein levels are highest in OPCs and decline as cells mature. TET1 protein can be detected in the oligodendroglial cytoplasm, the nucleus, and the cell processes. Unlike TET1, levels of TET2 remain constant. However, during initiation of the myelination process, TET2 translocates from the cytoplasm to the nucleus, indicating that TET2 activity is specifically needed for the wrapping process. TET3 remains localized to the nucleus throughout oligodendrogenesis. Of note, *in vitro* knockdown approaches for every member of TET family enzymes resulted in up-regulation of differentiation inhibitors such as *Hes1* or *Id2* and down-regulation of *MBP* and *PLP* gene expression [156]. In MS patients the expression levels of *TET2* in peripheral blood mononuclear cells was found to be significantly down-regulated and aberrant DNA hydroxymethylation resulted in decreased 5hmC levels, suggesting a relevant role in MS pathophysiology [157].

### 3.4. Nuclear Translocation of Membrane Proteins

As describe above, expression of inhibitory transcription factors Hes1/5 is controlled by Notch signaling. Of note and in contrast to many membrane receptors that transmit signals to the nucleus via complex cascades, Notch receptors can also act on gene expression by translocating themselves to the nucleus. Activation of the non-canonical Notch signaling pathway by the axonal F3/contactin, for example, triggers nuclear translocation of NICD and has been demonstrated to promote both generation as well as differentiation of oligodendroglial cells [143,158]. In this context, a report by Nakahara and colleagues suggests that inadequate nucleocytoplasmic transport in oligodendroglial cells affects MS progression by interfering with glial differentiation, is of particular interest [47]. TAT-interacting protein 30 kDa (TIP30) is a pro-apoptotic factor expressed at elevated levels within oligodendroglial cells in MS lesions. TIP30 has been found to inhibit nuclear transport of NICD and subsequently of Notch-mediated oligodendroglial differentiation and myelination [47,159]. Given that nuclear translocation of NICD is mediated by the nuclear transporter importin-β recognizing the NLS, TIP30 clearly inhibits differentiation as a direct inhibitor of importin-β [160,161]. Moreover, overexpression of TIP30 was found to sequester the transcription factor Olig1 in the cytoplasm and to weaken its nuclear translocation, whereas TIP30 knockdown enhanced nuclear Olig1 localization during the initiation stage of OPC differentiation [162].

The axon-derived ligand neuregulin 1 type III (NRG1) interacts with ErbB receptor tyrosine kinases expressed on oligodendroglial cells and affects their survival, maturation, and myelination [163,164,165] (Table 3). NRG binding provokes γ-secretase driven proteolytic cleavage and the release and nuclear translocation of ErbB4 intracellular domain (EICD), which influences target gene expression [166]. Nuclear accumulation of EICD has been shown to promote MBP expression, which can be blocked by inhibition of ErbB4 or γ-secretase [164]. After spinal cord injury (SCI), NRG1 signaling is decreased resulting in an inadequate ability of precursor cells to replenish oligodendrocytes [167]. However, NRG1 or ErbB gene depletion were found to have no effect on remyelination following LPC-induced demyelination, whereas overexpressing NRG1 resulted in axonal hypermyelination [168]. Furthermore, reduced ErbB4 expression in immune cells (total peripheral blood mononuclear cells, T cells, monocytes and B cells) of patients with relapsing remitting MS was observed, and suggests that insufficient ErbB signaling could also be associated with disease development [169].

Nuclear receptors belong to a superfamily that consists of a large number of ligand-activated transcription factors [170]. The superfamily encompasses two types of nuclear receptors. Type I receptors are classic steroid receptors that mediate the actions of steroid hormones such as glucocorticoids, mineralcorticoids, progestins, androgens, and estrogens. Type II receptors include thyroid hormone receptors (TRs), retinoid X receptors (RXRs), retinoic acid receptors (RARs), the receptor for 1,25-(OH)_2_ vitamin D_3_ (VDR), the peroxisome proliferator-activated receptors (PPARs), and many orphan receptors.

Upon ligand binding TRs as well as RARs exhibit nuclear localization and interact with the transcriptional machinery in the nucleus [170,171]. Furthermore, these nuclear receptors bind to specific promoter regions, thereby regulating transcriptional expression of *MBP*, *PLP*, *MAG*, and *2′,3′-cyclic nucleotide 3′-phosphodiesterase* [172]—hence promotes oligodendroglial cell differentiation [173].

RXRs are a family of nuclear receptors previously shown to be important regulators of differentiation [174] (Table 3). The RXR family comprises the receptors RXRα, RXRβ, and RXRγ, which form homodimers or heterodimers with other nuclear receptors, including TRs, RARs, VDRs, and PPARs to control transcription of target genes. Interestingly, strong expression of all three RXR members was observed in lesions following CNS injury [175]. During CNS remyelination RXRγ was differentially expressed in rodent oligodendroglial cells as well as in human acute and remyelinating MS lesions [176]. Depending on the oligodendroglial maturation state, RXRγ can either be found in the cytoplasm of Nkx2.2-positive OPCs or in the nucleus of mature oligodendrocytes as revealed by their expression of the maturation marker adenomatous polyposis coli APC/CC1-positive. Thus localization of RXRγ in the cytoplasm may inhibit oligodendroglial differentiation, whereas nuclear localization may promote the differentiation process [176]. In MS lesions, subcellular receptor localization correlated with the remyelination status as elevated nuclear RXRγ localization was observed in active remyelinating lesions compared to chronic inactive lesions [176].

The PPARs function as transcription factors to regulate target genes that are involved in lipid metabolism, and they have been shown to play a significant role in oligodendroglial differentiation [177,178,179] (Table 3). Upon ligand binding PPARs form heterodimers with RXR receptors. They can also translocate into the nucleus and interact with specific PPAR response elements (PPREs) in the promoter region of PPAR-target genes, resulting in enhanced MBP transcript levels as well as an increased numbers of O4-, O1-, and MBP-positive cells [179]. After spinal cord injury the number of PPAR+ oligodendroglial cells were found to be increased and to correlate in time and location with regions featuring robust oligodendrogenesis [180]. Furthermore, elevated expression of PPARγ has been observed during EAE in mice and PPARγ agonists were shown to ameliorate the disease course by inhibiting the expansion of encephalitogenic T cells [181]. Moreover, elevated levels of PPARγ have been observed in the cerebrospinal fluid of patients with MS [182]. The thromboxane A2 (TXA2) receptor (TPR) was found to be expressed in the developing rat brain during myelination, and expression levels increased during oligodendrocyte maturation [183]. During differentiation, TPR translocates from the cytoplasm in OPCs to the nucleus in oligodendrocytes, and receptor activation has been shown to result in CREB phosphorylation and to enhance MBP promoter activity [184].

**Table 3:** Nuclear translocation of membrane proteins.

Molecules	Major Function in OPCs	Role in MS/MS-Models	References
NRG	survival, differentiation, myelination	LPC, MS lesions: oligodendrogenesis, immune cells	Barres *et al.*, 1999 [165]; Fernandez *et al.*, 2000 [163]; Lai *et al.*, 2004 [164]; Brinkmann *et al.*, 2008 [168]; Tynyakov-Samra *et al.*, 2011 [169]; Gauthier *et al.*, 2013 [167]
RXRs	differentiation	LPC, MS lesions: remyelination	Schrage *et al.*, 2006 [175]; Huang *et al.*, 2011 [176]
PPARs	differentiation	SCI, EAE, MS lesions: oligodendrogenesis, immune cells	Saluja 2001 *et al.*, [177]; Woods *et al.*, 2003 [178]; Almad *et al.*, 2010 [180]; Bernardo *et al.*, 2013 [179]; Szalardy *et al.*, 2013 [182]; Unoda *et al.*, 2013 [181]

Abbreviation: lysolecithin (LPC), Multiple Sclerosis (MS), experimental autoimmune encephalomyelitis (EAE), spinal cord injury (SCI).

### 3.5. RNA Transport

Apart from a number of proteins for which the subcellular localizations is arranged during oligodendrogenesis, strong evidence also exists for the subcellular transport and distribution of selected RNA molecules. Notably, mRNAs for myelin proteins such as MBP are transported from the nucleus to terminal processes of oligodendroglial cells, where they are locally translated at the site of developing myelin sheaths [185]. In oligodendroglial cells, specific mRNA molecules are transported in RNA-protein complexes termed RNA granules that contain several essential molecules of the translational machinery including aminoacyl-tRNA synthetases, elongation factors and ribosomes [186]. RNA granules are carried along microtubules by the activity of kinesin and dynein motor proteins [187]. Before RNA granules reach their destination they are in a translationally silenced state and local translation becomes initiated upon specific signals [188,189]. Of note, in neurons retrograde transport and the activity of dynein binding proteins LIS1 and NDEL crucial for axonal transport are dependent on the activity of CDK5 [58]. Hence, decreased oligodendroglial maturation in the absence of CDK5 has been proposed to result from perturbed mRNA transport [57].

RNA granule formation, cargo selection, and cytoplasmic transport are tightly controlled by RNA-binding proteins referred to as the trans*-*acting factors heterogeneous nuclear ribonucleoproteins, including hnRNPA2, -A3 and quaking (QKI) [190,191]. Mutations in hnRNPs are thought to contribute to the pathogenesis of MS because some patients exhibit a single nucleotide variant in the hnRNPA1-encoding sequence leading to protein mislocalization and colocalization with stress granules and causing cellular apoptosis [192,193]. Stress granules (SGs) are aggregates of stalled translational preinitiation complexes that accumulate during stress. mRNAs in SGs are not translated as they do not contain 60S ribosomal subunits [194,195]. Thus, it is assumed that SGs store mRNA and ensure its sorting between translation and degradation [195]. The alternative splice isoform QKI-6 may be a component of SGs in oligodendroglial cells [191], and QKI-7 has been demonstrated to induce oligodendroglial apoptosis, whereas heterodimerization of QKI isoforms and subsequent nuclear translocation of QKI-7 can suppress apoptotic cell death [196].

However, MBP protein variants, resulting from different mRNA splice variants, have been shown to be differently distributed within oligodendroglial cells, depending on which isoforms are encoded by exons I-VII [197]. It was speculated that exon II MBP proteins may contain an NLS sequence and become actively transported through the NPC into the oligodendroglial nucleus [198]. Of note, nuclear MBP protein accumulation has been shown to influence cellular proliferation by elevating phosphorylation of ERK1/2 and Akt1 signaling proteins, indicating that secondary functions besides myelin sheath constitution and electrical isolation are encoded [197,199,200].

Finally, lamins-the structural components of the filamentous protein meshwork essential to inner nuclear membrane structure-are also functionally engaged in maturation processes. Overexpression of lamin B1 leads to a disturbance of inner nuclear membrane proteins and of chromatin organization, mislocalization of NPCs, and impaired nuclear pore transport [201,202]. As a consequence, premature arrest of oligodendroglial differentiation, due to reduced myelin transcription and misguided myelin proteins, is observed [202]. Interestingly, microRNA (miR)-dependent regulation, namely by miR-23, can contribute to this process [202].

## 4. Conclusions

**Figure 3:**
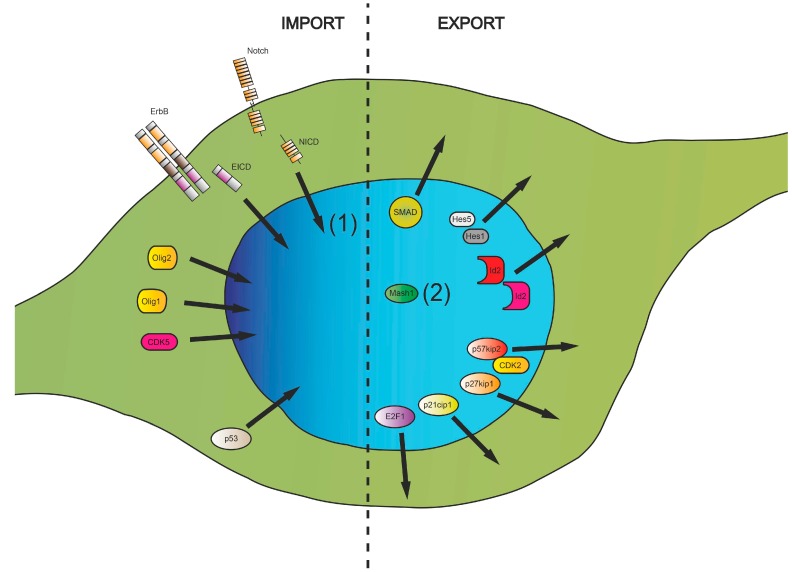
Summary of intracellular translocation directions of selected key proteins involved in transcriptional regulation, cell cycle control and transmission of cell surface signals revealed to be important for differentiation of immature oligodendroglial cells. (1) Nuclear import of Notch intracellular domain (NICD) was shown to have a different impact on the differentiation process depending on the nature of Notch ligands (Jagged1, F3/contactin); (2) The Mash1 protein is not exported from the nucleus; however, its inhibitory binding partner p57kip2 is actively translocated during the differentiation process thereby allowing Mash1 dependent transactivation of target genes to occur.

Since deregulated nucleocytoplasmic transport and the subsequent aberrant intracellular distribution of proteins appears to be critically involved in oligodendroglial differentiation failure and in myelin repair deficits (Figure 3), the identification of rate-limiting factors, either for export and/or import activities, might help to identify new target structures for future pharmacological remyelination therapies. In light of the growing number of immunomodulatory treatments approved for treating MS and the fact that no treatment options currently exist for damage repair, interest in the development of such therapies is increasing. It remains to be shown whether currently known proteins/(protein-complexes), as described here, are suitable targets for such strategies or whether superior (master) regulators need to be identified. Of note, recent studies in EAE mice revealed that oral administration of reversible CRM1 inhibitors could significantly attenuate disease progression [203]. Among effects on the immune cell compartment demyelinated axons appeared to be preserved by this treatment indicating that nucleocytoplasmic transport is also a critical process for neuroprotection. In light of this new data, it remains to be shown to what extent axonal regeneration/preservation and myelin repair, the two major deficits in the injured and diseased CNS, can simultaneously be promoted by pharmacological modulators of importins and exportins.

## References

[1] Waxman S.G. (1992). Demyelination in spinal cord injury and multiple sclerosis: What can we do to enhance functional recovery?. J. Neurotrauma.

[2] Trapp B.D., Peterson J., Ransohoff R.M., Rudick R., Mork S., Bo L. (1998). Axonal transection in the lesions of multiple sclerosis. N. Engl. J. Med..

[3] Bitsch A., Schuchardt J., Bunkowski S., Kuhlmann T., Bruck W. (2000). Acute axonal injury in multiple sclerosis. Correlation with demyelination and inflammation. Brain.

[4] Antel J., Antel S., Caramanos Z., Arnold D.L., Kuhlmann T. (2012). Primary progressive multiple sclerosis: Part of the MS disease spectrum or separate disease entity?. Acta Neuropathol..

[5] Love S. (2006). Demyelinating diseases. J. Clin. Pathol..

[6] Pomeroy I.M., Jordan E.K., Frank J.A., Matthews P.M., Esiri M.M. (2010). Focal and diffuse cortical degenerative changes in a marmoset model of multiple sclerosis. Mult. Scler..

[7] Wegner C., Esiri M.M., Chance S.A., Palace J., Matthews P.M. (2006). Neocortical neuronal, synaptic, and glial loss in multiple sclerosis. Neurology.

[8] Chang A., Nishiyama A., Peterson J., Prineas J., Trapp B.D. (2000). Ng2-positive oligodendrocyte progenitor cells in adult human brain and multiple sclerosis lesions. J. Neurosci..

[9] Ffrench-Constant C., Raff M.C. (1986). The oligodendrocyte-type-2 astrocyte cell lineage is specialized for myelination. Nature.

[10] Ffrench-Constant C., Raff M.C. (1986). Proliferating bipotential glial progenitor cells in adult rat optic nerve. Nature.

[11] Nishiyama A., Komitova M., Suzuki R., Zhu X. (2009). Polydendrocytes (Ng2 cells): Multifunctional cells with lineage plasticity. Nat. Rev. Neurosci..

[12] Levine J.M., Reynolds R., Fawcett J.W. (2001). The oligodendrocyte precursor cell in health and disease. Trends Neurosci..

[13] Levine J.M., Reynolds R. (1999). Activation and proliferation of endogenous oligodendrocyte precursor cells during ethidium bromide-induced demyelination. Exp. Neurol..

[14] Redwine J.M., Armstrong R.C. (1998). *In vivo* proliferation of oligodendrocyte progenitors expressing pdgfalphar during early remyelination. J. Neurobiol..

[15] Fancy S.P., Zhao C., Franklin R.J. (2004). Increased expression of Nkx2.2 and Olig2 identifies reactive oligodendrocyte progenitor cells responding to demyelination in the adult cns. Mol. Cell. Neurosci..

[16] Sim F.J., Zhao C., Penderis J., Franklin R.J. (2002). The age-related decrease in CNS remyelination efficiency is attributable to an impairment of both oligodendrocyte progenitor recruitment and differentiation. J. Neurosci..

[17] Arnett H.A., Fancy S.P., Alberta J.A., Zhao C., Plant S.R., Kaing S., Raine C.S., Rowitch D.H., Franklin R.J., Stiles C.D. (2004). bHLH transcription factor Olig1 is required to repair demyelinated lesions in the cns. Science.

[18] Watanabe M., Hadzic T., Nishiyama A. (2004). Transient up-regulation of Nkx2.2 expression in oligodendrocyte lineage cells during remyelination. Glia.

[19] Vana A.C., Lucchinetti C.F., Le T.Q., Armstrong R.C. (2007). Myelin transcription factor 1 (Myt1) expression in demyelinated lesions of rodent and human CNS. Glia.

[20] Aguirre A., Dupree J.L., Mangin J.M., Gallo V. (2007). A functional role for EGFR signaling in myelination and remyelination. Nat. Neurosci..

[21] Franklin R.J., Gallo V. (2014). The translational biology of remyelination: Past, present, and future. Glia.

[22] Bruce C.C., Zhao C., Franklin R.J. (2010). Remyelination—An effective means of neuroprotection. Horm. Behav..

[23] Franklin R.J., Ffrench-Constant C. (2008). Remyelination in the CNS: From biology to therapy. Nat. Rev. Neurosci..

[24] Rivera F.J., Steffenhagen C., Kremer D., Kandasamy M., Sandner B., Couillard-Despres S., Weidner N., Küry P., Aigner L. (2010). Deciphering the oligodendrogenic program of neural progenitors: Cell intrinsic and extrinsic regulators. Stem Cells Dev..

[25] Kremer D., Aktas O., Hartung H.P., Küry P. (2011). The complex world of oligodendroglial differentiation inhibitors. Ann. Neurol..

[26] Reynolds R., Dawson M., Papadopoulos D., Polito A., di Bello I.C., Pham-Dinh D., Levine J. (2002). The response of Ng2-expressing oligodendrocyte progenitors to demyelination in MOG-EAE and MS. J. Neurocytol..

[27] Franklin R.J., Kotter M.R. (2008). The biology of CNS remyelination: The key to therapeutic advances. J. Neurol..

[28] Blakemore W.F., Franklin R.J. (2008). Remyelination in experimental models of toxin-induced demyelination. Curr. Top. Microbiol. Immunol..

[29] Kuhlmann T., Miron V., Cui Q., Wegner C., Antel J., Bruck W. (2008). Differentiation block of oligodendroglial progenitor cells as a cause for remyelination failure in chronic multiple sclerosis. Brain.

[30] Chang A., Tourtellotte W.W., Rudick R., Trapp B.D. (2002). Premyelinating oligodendrocytes in chronic lesions of multiple sclerosis. N. Engl. J. Med..

[31] Wolswijk G. (1998). Chronic stage multiple sclerosis lesions contain a relatively quiescent population of oligodendrocyte precursor cells. J. Neurosci..

[32] Butler G.S., Overall C.M. (2009). Proteomic identification of multitasking proteins in unexpected locations complicates drug targeting. Nat. Rev. Drug Discov..

[33] Xu D., Farmer A., Chook Y.M. (2010). Recognition of nuclear targeting signals by karyopherin-β proteins. Curr. Opin. Struct. Biol..

[34] Mor A., White M.A., Fontoura B.M. (2014). Nuclear trafficking in health and disease. Curr. Opin. Cell Biol..

[35] Ossareh-Nazari B., Bachelerie F., Dargemont C. (1997). Evidence for a role of CRM1 in signal-mediated nuclear protein export. Science.

[36] Kosugi S., Hasebe M., Tomita M., Yanagawa H. (2008). Nuclear export signal consensus sequences defined using a localization-based yeast selection system. Traffic.

[37] Corbett A.H., Silver P.A. (1997). Nucleocytoplasmic transport of macromolecules. Microbiol. Mol. Biol. Rev..

[38] Gorlich D., Mattaj I.W. (1996). Nucleocytoplasmic transport. Science.

[39] Wente S.R., Rout M.P. (2010). The nuclear pore complex and nuclear transport. Cold Spring Harb. Perspect. Biol..

[40] Hung M.C., Link W. (2011). Protein localization in disease and therapy. J. Cell Sci..

[41] Dormann D., Rodde R., Edbauer D., Bentmann E., Fischer I., Hruscha A., Than M.E., Mackenzie I.R., Capell A., Schmid B. (2010). ALS-associated fused in sarcoma (FUS) mutations disrupt transportin-mediated nuclear import. EMBO J..

[42] Zhang J., Ito H., Wate R., Ohnishi S., Nakano S., Kusaka H. (2006). Altered distributions of nucleocytoplasmic transport-related proteins in the spinal cord of a mouse model of amyotrophic lateral sclerosis. Acta Neuropathol..

[43] Nagara Y., Tateishi T., Yamasaki R., Hayashi S., Kawamura M., Kikuchi H., Iinuma K.M., Tanaka M., Iwaki T., Matsushita T. (2013). Impaired cytoplasmic-nuclear transport of hypoxia-inducible factor-1α in amyotrophic lateral sclerosis. Brain Pathol..

[44] Sheffield L.G., Miskiewicz H.B., Tannenbaum L.B., Mirra S.S. (2006). Nuclear pore complex proteins in alzheimer disease. J. Neuropathol. Exp. Neurol..

[45] Ju T.C., Chen H.M., Lin J.T., Chang C.P., Chang W.C., Kang J.J., Sun C.P., Tao M.H., Tu P.H., Chang C. (2011). Nuclear translocation of AMPK-α1 potentiates striatal neurodegeneration in huntington’s disease. J. Cell Biol..

[46] Li A., Zou F., Fu H., Cui G., Yan Y., Wu Q., Gu X. (2013). Up-regulation of CRM1 relates to neuronal apoptosis after traumatic brain injury in adult rats. J. Mol. Neurosci. MN.

[47] Nakahara J., Kanekura K., Nawa M., Aiso S., Suzuki N. (2009). Abnormal expression of Tip30 and arrested nucleocytoplasmic transport within oligodendrocyte precursor cells in multiple sclerosis. J. Clin. Investig..

[48] Kim J.Y., Shen S., Dietz K., He Y., Howell O., Reynolds R., Casaccia P. (2010). HDAC1 nuclear export induced by pathological conditions is essential for the onset of axonal damage. Nat. Neurosci..

[49] Caillava C., Baron-Van Evercooren A. (2012). Differential requirement of cyclin-dependent kinase 2 for oligodendrocyte progenitor cell proliferation and differentiation. Cell Div..

[50] Ghiani C., Gallo V. (2001). Inhibition of cyclin e-cyclin-dependent kinase 2 complex formation and activity is associated with cell cycle arrest and withdrawal in oligodendrocyte progenitor cells. J. Neurosci..

[51] Frederick T.J., Min J., Altieri S.C., Mitchell N.E., Wood T.L. (2007). Synergistic induction of cyclin d1 in oligodendrocyte progenitor cells by IGF-I and FGF-2 requires differential stimulation of multiple signaling pathways. Glia.

[52] Jiang F., Frederick T.J., Wood T.L. (2001). IGF-I synergizes with FGF-2 to stimulate oligodendrocyte progenitor entry into the cell cycle. Dev. Biol..

[53] Malumbres M., Barbacid M. (2005). Mammalian cyclin-dependent kinases. Trends Biochem. Sci..

[54] Caillava C., Vandenbosch R., Jablonska B., Deboux C., Spigoni G., Gallo V., Malgrange B., Baron-Van Evercooren A. (2011). CDK2 loss accelerates precursor differentiation and remyelination in the adult central nervous system. J. Cell Biol..

[55] Göttle P., Sabo J.K., Heinen A., Venables G., Torres K., Tzekova N., Parras C.M., Kremer D., Hartung H.P., Cate H.S. (2015). Oligodendroglial maturation is dependent on intracellular protein shuttling. J. Neurosci..

[56] Zhou J., Li H., Li X., Zhang G., Niu Y., Yuan Z., Herrup K., Zhang Y.W., Bu G., Xu H. (2015). The roles of CDK5-mediated subcellular localization of FOXO1 in neuronal death. J. Neurosci..

[57] Yang Y., Wang H., Zhang J., Luo F., Herrup K., Bibb J.A., Lu R., Miller R.H. (2013). Cyclin dependent kinase 5 is required for the normal development of oligodendrocytes and myelin formation. Dev. Biol..

[58] Pandey J.P., Smith D.S. (2011). A CDK5-dependent switch regulates LIS1/NDEL1/dynein-driven organelle transport in adult axons. J. Neurosci..

[59] Miyamoto Y., Yamauchi J., Chan J.R., Okada A., Tomooka Y., Hisanaga S., Tanoue A. (2007). CDK5 regulates differentiation of oligodendrocyte precursor cells through the direct phosphorylation of paxillin. J. Cell Sci..

[60] Miyamoto Y., Yamauchi J., Tanoue A. (2008). CDK5 phosphorylation of WAVE2 regulates oligodendrocyte precursor cell migration through nonreceptor tyrosine kinase fyn. J. Neurosci..

[61] Sloane J.A., Vartanian T.K. (2007). WAVE1 and regulation of actin nucleation in myelination. Neurosci. Rev. J. Bringing Neurobiol. Neurol. Psychiatry.

[62] Martinez-Balbas M.A., Bauer U.M., Nielsen S.J., Brehm A., Kouzarides T. (2000). Regulation of E2F1 activity by acetylation. EMBO J..

[63] Dyson N. (1998). The regulation of E2F by prb-family proteins. Genes Dev..

[64] Weinberg R.A. (1995). The retinoblastoma protein and cell cycle control. Cell.

[65] Magri L., Swiss V.A., Jablonska B., Lei L., Pedre X., Walsh M., Zhang W., Gallo V., Canoll P., Casaccia P. (2014). E2F1 coregulates cell cycle genes and chromatin components during the transition of oligodendrocyte progenitors from proliferation to differentiation. J. Neurosci..

[66] Sherr C.J., Roberts J.M. (1999). CDK inhibitors: Positive and negative regulators of G1-phase progression. Genes Dev..

[67] Sherr C.J., Roberts J.M. (1995). Inhibitors of mammalian G1 cyclin-dependent kinases. Genes Dev..

[68] Casaccia-Bonnefil P., Tikoo R., Kiyokawa H., Friedrich V., Chao M.V., Koff A. (1997). Oligodendrocyte precursor differentiation is perturbed in the absence of the cyclin-dependent kinase inhibitor p27kip1. Genes Dev..

[69] Ghiani C.A., Eisen A.M., Yuan X., DePinho R.A., McBain C.J., Gallo V. (1999). Neurotransmitter receptor activation triggers p27(kip1)and p21(cip1) accumulation and G1 cell cycle arrest in oligodendrocyte progenitors. Development.

[70] Raff M. (2007). Intracellular developmental timers. Cold Spring Harb. Symp. Quant. Biol..

[71] Durand B., Gao F.B., Raff M. (1997). Accumulation of the cyclin-dependent kinase inhibitor p27/kip1 and the timing of oligodendrocyte differentiation. EMBO J..

[72] Miskimins R., Srinivasan R., Marin-Husstege M., Miskimins W.K., Casaccia-Bonnefil P. (2002). P27(kip1) enhances myelin basic protein gene promoter activity. J. Neurosci. Res..

[73] Tamaki S., Tokumoto Y. (2014). Over-expression of cyclin dependent kinase inhibitor p27/kip1 increases oligodendrocyte differentiation from induced pluripotent stem cells. In Vitro Cell. Dev. Biol. Anim..

[74] Durand B., Fero M.L., Roberts J.M., Raff M.C. (1998). P27kip1 alters the response of cells to mitogen and is part of a cell-intrinsic timer that arrests the cell cycle and initiates differentiation. Curr. Biol..

[75] Shen A., Liu Y., Zhao J., Qin J., Shi S., Chen M., Gao S., Xiao F., Lu Q., Cheng C. (2008). Temporal-spatial expressions of p27kip1 and its phosphorylation on serine-10 after acute spinal cord injury in adult rat: Implications for post-traumatic glial proliferation. Neurochem. Int..

[76] Crockett D.P., Burshteyn M., Garcia C., Muggironi M., Casaccia-Bonnefil P. (2005). Number of oligodendrocyte progenitors recruited to the lesioned spinal cord is modulated by the levels of the cell cycle regulatory protein p27kip-1. Glia.

[77] Eizenberg O., Faber-Elman A., Gottlieb E., Oren M., Rotter V., Schwartz M. (1996). P53 plays a regulatory role in differentiation and apoptosis of central nervous system-associated cells. Mol. Cell. Biol..

[78] Franklin D.S., Godfrey V.L., Lee H., Kovalev G.I., Schoonhoven R., Chen-Kiang S., Su L., Xiong Y. (1998). Cdk inhibitors p18(ink4c) and p27(kip1) mediate two separate pathways to collaboratively suppress pituitary tumorigenesis. Genes Dev..

[79] Tokumoto Y.M., Apperly J.A., Gao F.B., Raff M.C. (2002). Posttranscriptional regulation of p18 and p27 CDK inhibitor proteins and the timing of oligodendrocyte differentiation. Dev. Biol..

[80] Wosik K., Antel J., Kuhlmann T., Bruck W., Massie B., Nalbantoglu J. (2003). Oligodendrocyte injury in multiple sclerosis: A role for p53. J. Neurochem..

[81] Coqueret O. (2003). New roles for p21 and p27 cell-cycle inhibitors: A function for each cell compartment?. Trends Cell Biol..

[82] Besson A., Dowdy S.F., Roberts J.M. (2008). CDK inhibitors: Cell cycle regulators and beyond. Dev. Cell.

[83] Heinen A., Kremer D., Göttle P., Kruse F., Hasse B., Lehmann H., Hartung H.P., Küry P. (2008). The cyclin-dependent kinase inhibitor p57kip2 is a negative regulator of schwann cell differentiation and *in vitro* myelination. Proc. Natl. Acad. Sci. USA.

[84] Jadasz J.J., Rivera F.J., Taubert A., Kandasamy M., Sandner B., Weidner N., Aktas O., Hartung H.P., Aigner L., Küry P. (2012). p57kip2 regulates glial fate decision in adult neural stem cells. Development.

[85] Kremer D., Heinen A., Jadasz J., Göttle P., Zimmermann K., Zickler P., Jander S., Hartung H.P., Küry P. (2009). p57kip2 is dynamically regulated in experimental autoimmune encephalomyelitis and interferes with oligodendroglial maturation. Proc. Natl. Acad. Sci. USA.

[86] Lee M.H., Reynisdottir I., Massague J. (1995). Cloning of p57kip2, a cyclin-dependent kinase inhibitor with unique domain structure and tissue distribution. Genes Dev..

[87] Pateras I.S., Apostolopoulou K., Niforou K., Kotsinas A., Gorgoulis V.G. (2009). p57kip2: “Kip”ing the cell under control. Mol. Cancer Res..

[88] Yokoo T., Toyoshima H., Miura M., Wang Y., Iida K.T., Suzuki H., Sone H., Shimano H., Gotoda T., Nishimori S. (2003). p57kip2 regulates actin dynamics by binding and translocating lim-kinase 1 to the nucleus. J. Biol. Chem..

[89] Arber S., Barbayannis F.A., Hanser H., Schneider C., Stanyon C.A., Bernard O., Caroni P. (1998). Regulation of actin dynamics through phosphorylation of cofilin by lim-kinase. Nature.

[90] Pfeifenbring S., Metz I., Kremer D., Küry P., Hartung H.P., Brück W. (2013). Oligodendroglial lineage cells express nuclear p57kip2 in multiple sclerosis lesions. Glia.

[91] Li H., He Y., Richardson W.D., Casaccia P. (2009). Two-tier transcriptional control of oligodendrocyte differentiation. Curr. Opin. Neurobiol..

[92] Lu Q.R., Yuk D., Alberta J.A., Zhu Z., Pawlitzky I., Chan J., McMahon A.P., Stiles C.D., Rowitch D.H. (2000). Sonic hedgehog—Regulated oligodendrocyte lineage genes encoding BHLH proteins in the mammalian central nervous system. Neuron.

[93] Wu M., Hernandez M., Shen S., Sabo J.K., Kelkar D., Wang J., O’Leary R., Phillips G.R., Cate H.S., Casaccia P. (2012). Differential modulation of the oligodendrocyte transcriptome by sonic hedgehog and bone morphogenetic protein 4 via opposing effects on histone acetylation. J. Neurosci..

[94] Zhou Q., Wang S., Anderson D.J. (2000). Identification of a novel family of oligodendrocyte lineage-specific basic helix-loop-helix transcription factors. Neuron.

[95] Lu Q.R., Sun T., Zhu Z., Ma N., Garcia M., Stiles C.D., Rowitch D.H. (2002). Common developmental requirement for olig function indicates a motor neuron/oligodendrocyte connection. Cell.

[96] Takebayashi H., Nabeshima Y., Yoshida S., Chisaka O., Ikenaka K. (2002). The basic helix-loop-helix factor olig2 is essential for the development of motoneuron and oligodendrocyte lineages. Curr. Biol..

[97] Li H., Lu Y., Smith H.K., Richardson W.D. (2007). Olig1 and Sox10 interact synergistically to drive myelin basic protein transcription in oligodendrocytes. J. Neurosci..

[98] Xin M., Yue T., Ma Z., Wu F.F., Gow A., Lu Q.R. (2005). Myelinogenesis and axonal recognition by oligodendrocytes in brain are uncoupled in Olig1-null mice. J. Neurosci..

[99] Othman A., Frim D.M., Polak P., Vujicic S., Arnason B.G., Boullerne A.I. (2011). Olig1 is expressed in human oligodendrocytes during maturation and regeneration. Glia.

[100] Niu J., Mei F., Wang L., Liu S., Tian Y., Mo W., Li H., Lu Q.R., Xiao L. (2012). Phosphorylated Olig1 localizes to the cytosol of oligodendrocytes and promotes membrane expansion and maturation. Glia.

[101] Balabanov R., Popko B. (2005). Myelin repair: Developmental myelination redux?. Nat. Neurosci..

[102] Cheng T., Xue X., Fu J. (2015). Effect of olig1 on the development of oligodendrocytes and myelination in a neonatal rat pvl model induced by hypoxia-ischemia. Mol. Med. Rep..

[103] Fukuda S., Kondo T., Takebayashi H., Taga T. (2004). Negative regulatory effect of an oligodendrocytic bhlh factor Olig2 on the astrocytic differentiation pathway. Cell Death Differ..

[104] Setoguchi T., Kondo T. (2004). Nuclear export of Olig2 in neural stem cells is essential for ciliary neurotrophic factor-induced astrocyte differentiation. J. Cell Biol..

[105] Magnus T., Coksaygan T., Korn T., Xue H., Arumugam T.V., Mughal M.R., Eckley D.M., Tang S.C., Detolla L., Rao M.S. (2007). Evidence that nucleocytoplasmic Olig2 translocation mediates brain-injury-induced differentiation of glial precursors to astrocytes. J. Neurosci. Res..

[106] Parras C.M., Hunt C., Sugimori M., Nakafuku M., Rowitch D., Guillemot F. (2007). The proneural gene Mash1 specifies an early population of telencephalic oligodendrocytes. J. Neurosci..

[107] Sugimori M., Nagao M., Parras C.M., Nakatani H., Lebel M., Guillemot F., Nakafuku M. (2008). Ascl1 is required for oligodendrocyte development in the spinal cord. Development.

[108] Nakatani H., Martin E., Hassani H., Clavairoly A., Maire C.L., Viadieu A., Kerninon C., Delmasure A., Frah M., Weber M. (2013). Ascl1/Mash1 promotes brain oligodendrogenesis during myelination and remyelination. J. Neurosci..

[109] Gokhan S., Marin-Husstege M., Yung S.Y., Fontanez D., Casaccia-Bonnefil P., Mehler M.F. (2005). Combinatorial profiles of oligodendrocyte-selective classes of transcriptional regulators differentially modulate myelin basic protein gene expression. J. Neurosci..

[110] Kim J.G., Armstrong R.C., v Agoston D., Robinsky A., Wiese C., Nagle J., Hudson L.D. (1997). Myelin transcription factor 1 (Myt1) of the oligodendrocyte lineage, along with a closely related cchc zinc finger, is expressed in developing neurons in the mammalian central nervous system. J. Neurosci. Res..

[111] Armstrong R.C., Kim J.G., Hudson L.D. (1995). Expression of myelin transcription factor 1 (Myt1), a “zinc-finger” DNA-binding protein, in developing oligodendrocytes. Glia.

[112] Samanta J., Kessler J.A. (2004). Interactions between id and olig proteins mediate the inhibitory effects of BMP4 on oligodendroglial differentiation. Development.

[113] Chen Y., Wu H., Wang S., Koito H., Li J., Ye F., Hoang J., Escobar S.S., Gow A., Arnett H.A. (2009). The oligodendrocyte-specific G protein-coupled receptor GPR17 is a cell-intrinsic timer of myelination. Nat. Neurosci..

[114] Wang S., Sdrulla A., Johnson J.E., Yokota Y., Barres B.A. (2001). A role for the helix-loop-helix protein ID2 in the control of oligodendrocyte development. Neuron.

[115] Lecca D., Trincavelli M.L., Gelosa P., Sironi L., Ciana P., Fumagalli M., Villa G., Verderio C., Grumelli C., Guerrini U. (2008). The recently identified p2y-like receptor GPR17 is a sensor of brain damage and a new target for brain repair. PLoS ONE.

[116] Shimizu T., Bae Y.K., Muraoka O., Hibi M. (2005). Interaction of Wnt and caudal-related genes in zebrafish posterior body formation. Dev. Biol..

[117] Fancy S.P., Baranzini S.E., Zhao C., Yuk D.I., Irvine K.A., Kaing S., Sanai N., Franklin R.J., Rowitch D.H. (2009). Dysregulation of the wnt pathway inhibits timely myelination and remyelination in the mammalian cns. Genes Dev..

[118] Lurbke A., Hagemeier K., Cui Q.L., Metz I., Bruck W., Antel J., Kuhlmann T. (2013). Limited Tcf7l2 expression in MS lesions. PLoS ONE.

[119] Cadigan K.M., Liu Y.I. (2006). Wnt signaling: Complexity at the surface. J. Cell Sci..

[120] Malbon C.C., Wang H.Y. (2006). Dishevelled: A mobile scaffold catalyzing development. Curr. Top. Dev. Biol..

[121] Azim K., Butt A.M. (2011). Gsk3beta negatively regulates oligodendrocyte differentiation and myelination *in vivo*. Glia.

[122] Feigenson K., Reid M., See J., Crenshaw E.B., Grinspan J.B. (2009). Wnt signaling is sufficient to perturb oligodendrocyte maturation. Mol. Cell. Neurosci..

[123] Ye F., Chen Y., Hoang T., Montgomery R.L., Zhao X.H., Bu H., Hu T., Taketo M.M., van Es J.H., Clevers H. (2009). HDAC1 and HDAC2 regulate oligodendrocyte differentiation by disrupting the β-catenin-tcf interaction. Nat. Neurosci..

[124] Fancy S.P., Harrington E.P., Yuen T.J., Silbereis J.C., Zhao C., Baranzini S.E., Bruce C.C., Otero J.J., Huang E.J., Nusse R. (2011). Axin2 as regulatory and therapeutic target in newborn brain injury and remyelination. Nat. Neurosci..

[125] Daniels D.L., Weis W.I. (2005). Beta-catenin directly displaces groucho/tle repressors from TCF/LEF in Wnt-mediated transcription activation. Nat. Struct. Mol. Biol..

[126] Labbe E., Letamendia A., Attisano L. (2000). Association of SMADs with lymphoid enhancer binding factor 1/t cell-specific factor mediates cooperative signaling by the transforming growth factor-β and wnt pathways. Proc. Natl. Acad. Sci. USA.

[127] See J.M., Grinspan J.B. (2009). Sending mixed signals: Bone morphogenetic protein in myelination and demyelination. J. Neuropathol. Exp. Neurol..

[128] Massague J. (1998). TGF-β signal transduction. Annu. Rev. Biochem..

[129] Kondo T., Raff M.C. (2004). A role for noggin in the development of oligodendrocyte precursor cells. Dev. Biol..

[130] Grinspan J.B., Edell E., Carpio D.F., Beesley J.S., Lavy L., Pleasure D., Golden J.A. (2000). Stage-specific effects of bone morphogenetic proteins on the oligodendrocyte lineage. J. Neurobiol..

[131] Sabo J.K., Aumann T.D., Merlo D., Kilpatrick T.J., Cate H.S. (2011). Remyelination is altered by bone morphogenic protein signaling in demyelinated lesions. J. Neurosci..

[132] Wang Y., Cheng X., He Q., Zheng Y., Kim D.H., Whittemore S.R., Cao Q.L. (2011). Astrocytes from the contused spinal cord inhibit oligodendrocyte differentiation of adult oligodendrocyte precursor cells by increasing the expression of bone morphogenetic proteins. J. Neurosci..

[133] Setoguchi T., Nakashima K., Takizawa T., Yanagisawa M., Ochiai W., Okabe M., Yone K., Komiya S., Taga T. (2004). Treatment of spinal cord injury by transplantation of fetal neural precursor cells engineered to express BMP inhibitor. Exp. Neurol..

[134] Fuller M.L., DeChant A.K., Rothstein B., Caprariello A., Wang R., Hall A.K., Miller R.H. (2007). Bone morphogenetic proteins promote gliosis in demyelinating spinal cord lesions. Ann. Neurol..

[135] Ara J., See J., Mamontov P., Hahn A., Bannerman P., Pleasure D., Grinspan J.B. (2008). Bone morphogenetic proteins 4, 6, and 7 are up-regulated in mouse spinal cord during experimental autoimmune encephalomyelitis. J. Neurosci. Res..

[136] Deininger M., Meyermann R., Schluesener H. (1995). Detection of two transforming growth factor-β-related morphogens, bone morphogenetic proteins-4 and -5, in RNA of multiple sclerosis and creutzfeldt-jakob disease lesions. Acta Neuropathol..

[137] Nakashima K., Yanagisawa M., Arakawa H., Kimura N., Hisatsune T., Kawabata M., Miyazono K., Taga T. (1999). Synergistic signaling in fetal brain by Stat3-Smad1 complex bridged by p300. Science.

[138] Massague J., Wotton D. (2000). Transcriptional control by the TGF-β/smad signaling system. EMBO J..

[139] Massague J., Seoane J., Wotton D. (2005). Smad transcription factors. Genes Dev..

[140] Ming M., Manzini I., Le W., Krieglstein K., Spittau B. (2010). Thapsigargin-induced Ca^2+^ increase inhibits TGFβ1-mediated Smad2 transcriptional responses via Ca^2+^/calmodulin-dependent protein kinase II. J. Cell. Biochem..

[141] Jarriault S., le Bail O., Hirsinger E., Pourquie O., Logeat F., Strong C.F., Brou C., Seidah N.G., Isra l A. (1998). Delta-1 activation of Notch-1 signaling results in HES-1 transactivation. Mol. Cell. Biol..

[142] Wang S., Sdrulla A.D., diSibio G., Bush G., Nofziger D., Hicks C., Weinmaster G., Barres B.A. (1998). Notch receptor activation inhibits oligodendrocyte differentiation. Neuron.

[143] Hu Q.D., Ang B.T., Karsak M., Hu W.P., Cui X.Y., Duka T., Takeda Y., Chia W., Sankar N., Ng Y.K. (2003). F3/contactin acts as a functional ligand for notch during oligodendrocyte maturation. Cell.

[144] John G.R., Shankar S.L., Shafit-Zagardo B., Massimi A., Lee S.C., Raine C.S., Brosnan C.F. (2002). Multiple sclerosis: Re-expression of a developmental pathway that restricts oligodendrocyte maturation. Nat. Med..

[145] Elyaman W., Bradshaw E.M., Wang Y., Oukka M., Kivisakk P., Chiba S., Yagita H., Khoury S.J. (2007). Jagged1 and delta1 differentially regulate the outcome of experimental autoimmune encephalomyelitis. J. Immunol..

[146] Tsugane S., Takizawa S., Kaneyama T., Ichikawa M., Yagita H., Kim B.S., Koh C.S. (2012). Therapeutic effects of anti-δ1 mab on theiler’s murine encephalomyelitis virus-induced demyelinating disease. J. Neuroimmunol..

[147] D’Souza B., Miyamoto A., Weinmaster G. (2008). The many facets of Notch ligands. Oncogene.

[148] Liu A., Li J., Marin-Husstege M., Kageyama R., Fan Y., Gelinas C., Casaccia-Bonnefil P. (2006). A molecular insight of HES5-dependent inhibition of myelin gene expression: Old partners and new players. EMBO J..

[149] Galiova G., Bartova E., Raska I., Krejci J., Kozubek S. (2008). Chromatin changes induced by lamin a/c deficiency and the histone deacetylase inhibitor trichostatin A. Eur. J. Cell Biol..

[150] Shen S., Sandoval J., Swiss V.A., Li J., Dupree J., Franklin R.J., Casaccia-Bonnefil P. (2008). Age-dependent epigenetic control of differentiation inhibitors is critical for remyelination efficiency. Nat. Neurosci..

[151] Camelo S., Iglesias A.H., Hwang D., Due B., Ryu H., Smith K., Gray S.G., Imitola J., Duran G., Assaf B. (2005). Transcriptional therapy with the histone deacetylase inhibitor trichostatin a ameliorates experimental autoimmune encephalomyelitis. J. Neuroimmunol..

[152] Faraco G., Cavone L., Chiarugi A. (2011). The therapeutic potential of hdac inhibitors in the treatment of multiple sclerosis. Mol. Med..

[153] Pedre X., Mastronardi F., Bruck W., Lopez-Rodas G., Kuhlmann T., Casaccia P. (2011). Changed histone acetylation patterns in normal-appearing white matter and early multiple sclerosis lesions. J. Neurosci..

[154] Mastronardi F.G., Wood D.D., Mei J., Raijmakers R., Tseveleki V., Dosch H.M., Probert L., Casaccia-Bonnefil P., Moscarello M.A. (2006). Increased citrullination of histone h3 in multiple sclerosis brain and animal models of demyelination: A role for tumor necrosis factor-induced peptidylarginine deiminase 4 translocation. J. Neurosci..

[155] Wu H., D’Alessio A.C., Ito S., Wang Z., Cui K., Zhao K., Sun Y.E., Zhang Y. (2011). Genome-wide analysis of 5-hydroxymethylcytosine distribution reveals its dual function in transcriptional regulation in mouse embryonic stem cells. Genes Dev..

[156] Zhao X., Dai J., Ma Y., Mi Y., Cui D., Ju G., Macklin W.B., Jin W. (2014). Dynamics of ten-eleven translocation hydroxylase family proteins and 5-hydroxymethylcytosine in oligodendrocyte differentiation. Glia.

[157] Calabrese R., Valentini E., Ciccarone F., Guastafierro T., Bacalini M.G., Ricigliano V.A., Zampieri M., Annibali V., Mechelli R., Franceschi C. (2014). Tet2 gene expression and 5-hydroxymethylcytosine level in multiple sclerosis peripheral blood cells. Biochim. Biophys. Acta.

[158] Cui X.Y., Hu Q.D., Tekaya M., Shimoda Y., Ang B.T., Nie D.Y., Sun L., Hu W.P., Karsak M., Duka T. (2004). Nb-3/Notch1 pathway via deltex1 promotes neural progenitor cell differentiation into oligodendrocytes. J. Biol. Chem..

[159] Brosnan C.F., John G.R. (2009). Revisiting Notch in remyelination of multiple sclerosis lesions. J. Clin. Investig..

[160] El Omari K., Bird L.E., Nichols C.E., Ren J., Stammers D.K. (2005). Crystal structure of cc3 (Tip30): Implications for its role as a tumor suppressor. J. Biol. Chem..

[161] King F.W., Shtivelman E. (2004). Inhibition of nuclear import by the proapoptotic protein cc3. Mol. Cell. Biol..

[162] Yang W., Xiao L., Li C., Liu X., Liu M., Shao Q., Wang D., Huang A., He C. (2015). Tip30 inhibits oligodendrocyte precursor cell differentiation via cytoplasmic sequestration of Olig1. Glia.

[163] Fernandez P.A., Tang D.G., Cheng L., Prochiantz A., Mudge A.W., Raff M.C. (2000). Evidence that axon-derived neuregulin promotes oligodendrocyte survival in the developing rat optic nerve. Neuron.

[164] Lai C., Feng L. (2004). Implication of γ-secretase in neuregulin-induced maturation of oligodendrocytes. Biochem. Biophys. Res. Commun..

[165] Barres B.A., Raff M.C. (1999). Axonal control of oligodendrocyte development. J. Cell Biol..

[166] Ni C.Y., Murphy M.P., Golde T.E., Carpenter G. (2001). Gamma-secretase cleavage and nuclear localization of ErbB-4 receptor tyrosine kinase. Science.

[167] Gauthier M.K., Kosciuczyk K., Tapley L., Karimi-Abdolrezaee S. (2013). Dysregulation of the neuregulin-1-ErbB network modulates endogenous oligodendrocyte differentiation and preservation after spinal cord injury. Eur. J. Neurosci..

[168] Brinkmann B.G., Agarwal A., Sereda M.W., Garratt A.N., Muller T., Wende H., Stassart R.M., Nawaz S., Humml C., Velanac V. (2008). Neuregulin-1/ErbB signaling serves distinct functions in myelination of the peripheral and central nervous system. Neuron.

[169] Tynyakov-Samra E., Auriel E., Levy-Amir Y., Karni A. (2011). Reduced ErbB4 expression in immune cells of patients with relapsing remitting multiple sclerosis. Mult. Scler. Int..

[170] Li D., Yamada T., Wang F., Vulin A.I., Samuels H.H. (2004). Novel roles of retinoid X receptor (RXR) and RXR ligand in dynamically modulating the activity of the thyroid hormone receptor/RXR heterodimer. J. Biol. Chem..

[171] Zhu X.G., Hanover J.A., Hager G.L., Cheng S.Y. (1998). Hormone-induced translocation of thyroid hormone receptors in living cells visualized using a receptor green fluorescent protein chimera. J. Biol. Chem..

[172] Oppenheimer J.H., Schwartz H.L. (1997). Molecular basis of thyroid hormone-dependent brain development. Endocr. Rev..

[173] Barres B.A., Lazar M.A., Raff M.C. (1994). A novel role for thyroid hormone, glucocorticoids and retinoic acid in timing oligodendrocyte development. Development.

[174] Lefebvre P., Benomar Y., Staels B. (2010). Retinoid x receptors: Common heterodimerization partners with distinct functions. Trends Endocrinol. Metab..

[175] Schrage K., Koopmans G., Joosten E.A., Mey J. (2006). Macrophages and neurons are targets of retinoic acid signaling after spinal cord contusion injury. Eur. J. Neurosci..

[176] Huang J.K., Jarjour A.A., Nait Oumesmar B., Kerninon C., Williams A., Krezel W., Kagechika H., Bauer J., Zhao C., Evercooren A.B. (2011). Retinoid X receptor γ signaling accelerates CNS remyelination. Nat. Neurosci..

[177] Saluja I., Granneman J.G., Skoff R.P. (2001). PPAR δ agonists stimulate oligodendrocyte differentiation in tissue culture. Glia.

[178] Woods J.W., Tanen M., Figueroa D.J., Biswas C., Zycband E., Moller D.E., Austin C.P., Berger J.P. (2003). Localization of ppardelta in murine central nervous system: Expression in oligodendrocytes and neurons. Brain Res..

[179] Bernardo A., de Simone R., de Nuccio C., Visentin S., Minghetti L. (2013). The nuclear receptor peroxisome proliferator-activated receptor-γ promotes oligodendrocyte differentiation through mechanisms involving mitochondria and oscillatory Ca^2+^ waves. Biol. Chem..

[180] Almad A., McTigue D.M. (2010). Chronic expression of PPAR-δ by oligodendrocyte lineage cells in the injured rat spinal cord. J. Comp. Neurol..

[181] Unoda K., Doi Y., Nakajima H., Yamane K., Hosokawa T., Ishida S., Kimura F., Hanafusa T. (2013). Eicosapentaenoic acid (EPA) induces peroxisome proliferator-activated receptors and ameliorates experimental autoimmune encephalomyelitis. J. Neuroimmunol..

[182] Szalardy L., Zadori D., Tanczos E., Simu M., Bencsik K., Vecsei L., Klivenyi P. (2013). Elevated levels of PPAR-γ in the cerebrospinal fluid of patients with multiple sclerosis. Neurosci. Lett..

[183] Ramamurthy S., Mir F., Gould R.M., le Breton G.C. (2006). Characterization of thromboxane a2 receptor signaling in developing rat oligodendrocytes: Nuclear receptor localization and stimulation of myelin basic protein expression. J. Neurosci. Res..

[184] Mir F., le Breton G.C. (2008). A novel nuclear signaling pathway for thromboxane a2 receptors in oligodendrocytes: Evidence for signaling compartmentalization during differentiation. Mol. Cell. Biol..

[185] Boccaccio G.L., Carminatti H., Colman D.R. (1999). Subcellular fractionation and association with the cytoskeleton of messengers encoding myelin proteins. J. Neurosci. Res..

[186] Barbarese E., Koppel D.E., Deutscher M.P., Smith C.L., Ainger K., Morgan F., Carson J.H. (1995). Protein translation components are colocalized in granules in oligodendrocytes. J. Cell Sci..

[187] Carson J.H., Kwon S., Barbarese E. (1998). RNA trafficking in myelinating cells. Curr. Opin. Neurobiol..

[188] Czaplinski K., Singer R.H. (2006). Pathways for mRNA localization in the cytoplasm. Trends Biochem. Sci..

[189] Kiebler M.A., Bassell G.J. (2006). Neuronal RNA granules: Movers and makers. Neuron.

[190] White R., Gonsior C., Bauer N.M., Kramer-Albers E.M., Luhmann H.J., Trotter J. (2012). Heterogeneous nuclear ribonucleoprotein (hnRNP) F is a novel component of oligodendroglial rna transport granules contributing to regulation of myelin basic protein (MBP) synthesis. J. Biol. Chem..

[191] Wang Y., Lacroix G., Haines J., Doukhanine E., Almazan G., Richard S. (2010). The QKI-6 RNA binding protein localizes with the MBP mRNAs in stress granules of Glial cells. PLoS ONE.

[192] Lee S., Levin M. (2014). Novel somatic single nucleotide variants within the RNA binding protein hnRNP A1 in multiple sclerosis patients. F1000Research.

[193] Han S.P., Friend L.R., Carson J.H., Korza G., Barbarese E., Maggipinto M., Hatfield J.T., Rothnagel J.A., Smith R. (2010). Differential subcellular distributions and trafficking functions of hnRNP A2/B1 spliceoforms. Traffic.

[194] Kedersha N., Stoecklin G., Ayodele M., Yacono P., Lykke-Andersen J., Fritzler M.J., Scheuner D., Kaufman R.J., Golan D.E., Anderson P. (2005). Stress granules and processing bodies are dynamically linked sites of mrnp remodeling. J. Cell Biol..

[195] Nadezhdina E.S., Lomakin A.J., Shpilman A.A., Chudinova E.M., Ivanov P.A. (2010). Microtubules govern stress granule mobility and dynamics. Biochim. Biophys. Acta.

[196] Pilotte J., Larocque D., Richard S. (2001). Nuclear translocation controlled by alternatively spliced isoforms inactivates the quaking apoptotic inducer. Genes Dev..

[197] Ozgen H., Kahya N., de Jonge J.C., Smith G.S., Harauz G., Hoekstra D., Baron W. (2014). Regulation of cell proliferation by nucleocytoplasmic dynamics of postnatal and embryonic exon-II-containing MBP isoforms. Biochim. Biophys. Acta.

[198] Pedraza L., Fidler L., Staugaitis S.M., Colman D.R. (1997). The active transport of myelin basic protein into the nucleus suggests a regulatory role in myelination. Neuron.

[199] Smith G.S., Paez P.M., Spreuer V., Campagnoni C.W., Boggs J.M., Campagnoni A.T., Harauz G. (2011). Classical 18.5-and 21.5-kDa isoforms of myelin basic protein inhibit calcium influx into oligodendroglial cells, in contrast to golli isoforms. J. Neurosci. Res..

[200] Smith G.S., Samborska B., Hawley S.P., Klaiman J.M., Gillis T.E., Jones N., Boggs J.M., Harauz G. (2013). Nucleus-localized 21.5-kDa myelin basic protein promotes oligodendrocyte proliferation and enhances neurite outgrowth in coculture, unlike the plasma membrane-associated 18.5-kDa isoform. J. Neurosci. Res..

[201] Lin S.T., Heng M.Y., Ptacek L.J., Fu Y.H. (2014). Regulation of myelination in the central nervous system by nuclear lamin b1 and non-coding rnas. Transl. Neurodegener..

[202] Lin S.T., Fu Y.H. (2009). MIR-23 regulation of lamin b1 is crucial for oligodendrocyte development and myelination. Dis. Models Mech..

[203] Haines J.D., Herbin O., de la Hera B., Vidaurre O.G., Moy G.A., Sun Q., Fung H.Y., Albrecht S., Alexandropoulos K., McCauley D. (2015). Nuclear export inhibitors avert progression in preclinical models of inflammatory demyelination. Nat. Neurosci..

